# Newcastle disease virus fusion and haemagglutinin-neuraminidase proteins contribute to its macrophage host range

**DOI:** 10.1099/vir.0.048579-0

**Published:** 2013-06

**Authors:** Ingrid Cornax, Diego G. Diel, Cary A. Rue, Carlos Estevez, Qingzhong Yu, Patti J. Miller, Claudio L. Afonso

**Affiliations:** Southeast Poultry Research Laboratory, Agricultural Research Service, United States Department of Agriculture, Athens, GA 30605, USA

## Abstract

The fusion (F) and haemagglutinin-neuraminidase (HN) proteins of *Newcastle disease* *virus* (NDV) are multifunctional proteins that play critical roles during infection. Here, we assessed the ability of NDV to replicate in macrophages and investigated the contribution of the F and HN proteins to NDV infection/replication in these cells. Results of our study revealed that, while presenting similar replication kinetics in a fibroblast cell line (DF1) or in primary non-adherent splenocytes, the NDV strain CA02 replicates better in macrophages (HD11 and primary adherent splenocytes) than the NDV strain Anhinga/93. Notably, exchange of the HN or both F and HN genes of NDV Anhinga/93 by the corresponding genes from NDV CA02 markedly improved the ability of the chimeric viruses to replicate in macrophages. These results indicate that the F and HN proteins are determinants of NDV macrophage host range. This represents the first description of productive NDV infection in macrophages.

## Introduction

*Newcastle disease virus* (NDV) is an enveloped negative sense ssRNA virus of the genus *Avulavirus*, family *Paramyxoviridae* (Alexander & Senne, 2008). The NDV genome is ~15.2 kb in length and contains genes encoding at least seven proteins, including the nucleoprotein (NP), the phosphoprotein (P), the matrix protein (M), the fusion protein (F), the haemagglutinin-neuraminidase (HN), the RNA dependent RNA polymerase (L), and the V protein (Alexander & Senne, 2008). NDV infections result in a broad range of clinical manifestations, depending on the virulence of the virus isolate and the species of bird affected (Miller & Afonso, 2011).

Infection of host cells by NDV is mediated by two surface glycoproteins, the attachment (HN) protein and the fusion (F) protein (Chang & Dutch, 2012). The HN protein mediates virus attachment to sialic acid-containing receptors in the cell surface, functions as a neuraminidase by removing sialic acid molecules from progeny virions to prevent self-aggregation during budding, and promotes the fusion activity of the F protein. The F protein, on the other hand, directs the membrane fusion between the virus envelope and the cell membrane (Chang & Dutch, 2012; Morrison, 2003). This protein is synthesized as a precursor F0, which is cleaved into the active subunits F1 and F2. Cleavage of the precursor F0 alone, however, is not sufficient for fusion and the HN protein is also required for an efficient fusion process (Gotoh *et al.*, 1992; Scheid & Choppin, 1974). Because the F and HN proteins mediate fundamental aspects of NDV infection, they play important roles in virus virulence and tissue tropism (de Leeuw *et al.*, 2005; Dortmans *et al.*, 2011; Huang *et al.*, 2004; Panda *et al.*, 2004).

Several studies have shown that the amino acid composition of the F protein cleavage site is the main determinant of NDV virulence and tissue tropism (de Leeuw *et al.*, 2005; Dortmans *et al.*, 2011; Panda *et al.*, 2004). Virulent strains of NDV (velogenic and mesogenic) contain a multi-basic amino acid F protein cleavage site, which is cleaved by ubiquitous proteases expressed in a wide range of host cells and tissues (Dortmans *et al.*, 2011). In contrast, the F protein cleavage site of low virulence NDV strains (lentogenic) are usually mono-basic and are cleaved by trypsin-like enzymes which are present only in the respiratory and intestinal tracts (Dortmans *et al.*, 2011). Consequently, infections with virulent strains of NDV result in systemic often fatal disease, while infections with low virulence strains are usually restricted to the respiratory and digestive systems (Alexander & Senne, 2008).

Pathogenesis studies performed in chickens and chicken embryos have shown that the HN protein also functions as a virulence and host range determinant for NDV (Huang *et al.*, 2004; Khattar *et al.*, 2009). For example, when the HN protein of a low virulence NDV strain was exchanged with that of a virulent strain the chimeric viruses reached systemic sites, whereas the parental low virulence virus replicated only locally in the respiratory tract (Huang *et al.*, 2004). These observations suggest that the HN protein may contribute to NDV infection and replication in cells responsible for systemic dissemination of the virus.

*In vivo* studies have shown that NDV exhibits a marked tropism for lymphoid tissues, with viral antigen being frequently detected in tissue macrophages (Brown *et al.*, 1999). Macrophages are thought to be one of the main target cells for NDV *in vivo*; however, there is no convincing evidence demonstrating the ability of NDV to replicate in these cells. In the present study, we assessed the ability of different NDV strains to replicate in chicken macrophages and investigated the contribution of the surface glycoproteins (F and HN) for NDV infection/replication in these cells. Replication kinetics of parental wt NDV strains and chimeric viruses in which the HN or the F and HN genes have been exchanged were compared in chicken macrophages and chicken fibroblasts *in vitro*.

## Results

### NDV strain Anhinga/93 presents an impaired ability to replicate in macrophages *in vitro*

**Fig. 1.**
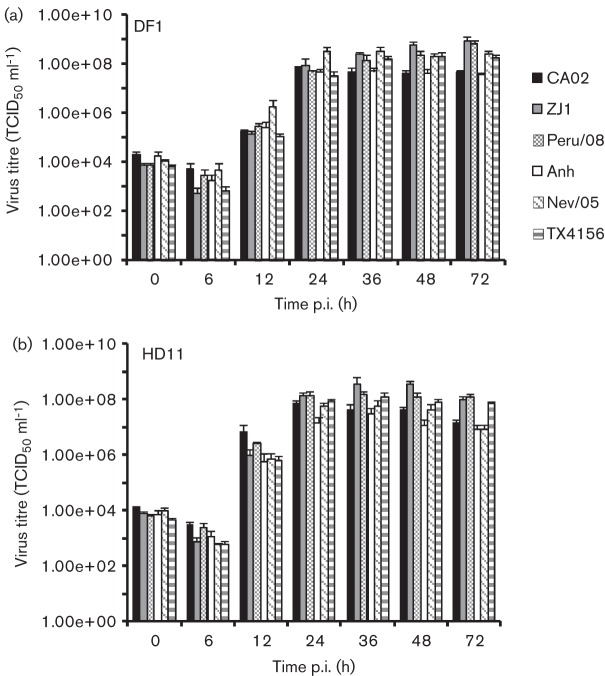
Replication kinetics of NDV strains CA02, ZJ1, Peru/08, Anhinga/93 (Anh), Nevada/05 (Nev/05) and TX4156. Multi-step growth curve in (a) DF1 chicken fibroblast cell line and (b) HD11 chicken macrophage cell line. Cells (a, b) were inoculated with each virus (m.o.i. = 0.01), and the supernatant (200 µl) was harvested at the indicated time points. Virus titres were determined by limiting dilution, calculated according to the Spearman and Karber method and expressed as log_10_ (TCID_50_ ml^−1^). Results shown are the mean±se of three independent experiments.

The ability of velogenic and mesogenic NDV strains to replicate in chicken macrophages was investigated *in vitro*. Replication characteristics of velogenic NDV strains CA02, ZJ1 and Peru/08 and mesogenic NDV strains Anhinga/93, Nevada/05 and TX4156 were assessed by multi-step growth curves in chicken fibroblast (DF1) and macrophage (HD11) cell lines (Diel *et al*., 2012; Liu *et al.*, 2007; Wakamatsu *et al.*, 2006; Estevez *et al.*, 2007; Susta *et al.*, 2011). DF1 and HD11 cells were cultured in six-well plates (10^6^ cells per well) and inoculated with each virus at an m.o.i. of 0.01, and the viral titres were determined by limiting dilution at 0, 6, 12, 24, 36, 48 and 72 h post-infection (p.i.). While all NDV strains replicated at similar levels in DF1 cells (Fig. 1a), NDV strain Anhinga/93 presented reduced viral yields in chicken macrophages (HD11) when compared with the other NDV strains (~0.5–1 log; Fig. 1b).

**Fig. 2.**
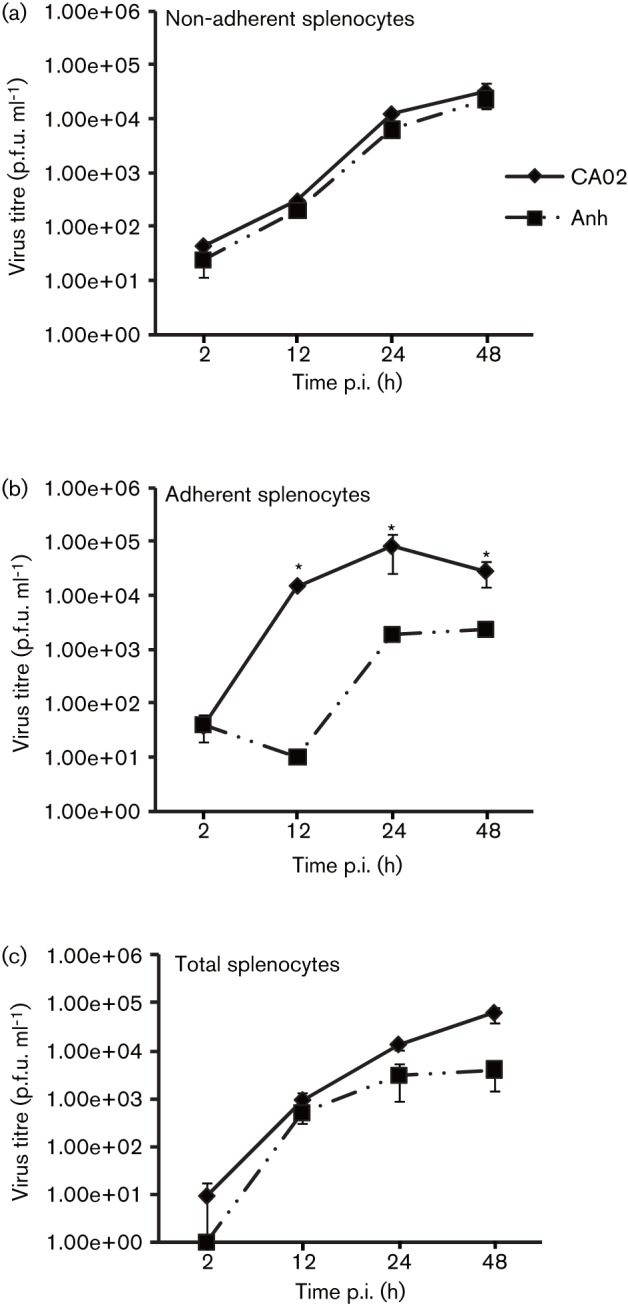
Replication kinetics of NDV strains CA02 and Anhinga/93 in primary splenocytes. Multi-step growth curve of NDV strains CA02 and Anhinga/93 (Anh) in (a) primary lymphocyte cell cultures (non-adherent splenocytes), (b) primary macrophage cultures (adherent splenocytes) and (c) primary splenocyte cell cultures (total splenocytes). Cells (a, b, c) were inoculated with each virus (m.o.i. = 0.01) and harvested at 2, 12, 24 and 48 h p.i. Virus titres were determined by plaque assay and expressed as p.f.u. ml^−1^. Results shown are the mean±se of three independent experiments. *P<0.02.

The replication kinetics of NDV strains CA02 and Anhinga/93, which presented similar growth characteristics in control DF1 cells (Fig. 1a), were further assessed in primary macrophages. Splenocytes were obtained from specific pathogen free (SPF) chickens using standard protocols, and three cell populations were separated: (1) total splenocytes; (2) non-adherent splenocytes (predominantly lymphocytes); and (3) adherent splenocytes (predominantly macrophages). Each cell type was cultured in 12-well plates (10^4^ cells per well) and inoculated with NDV strains CA02 and Anhinga/93 at an m.o.i. of 0.01, and viral titres were determined by plaque assay at 2, 12, 24 and 48 h p.i. (Huang *et al.*, 2004). No differences in replication kinetics or viral yields were observed between the two NDV strains in non-adherent splenocytes (lymphocytes; Fig. 2a); however, NDV strain Anhinga/93 presented a decreased ability to replicate in adherent (macrophages) and total (lymphocytes + macrophages) splenocytes as evidenced by altered replication kinetics (Fig. 2b) and/or lower viral yields (Fig. 2b, c). These results suggest that NDV strain Anhinga/93 has an impaired ability to infect and/or replicate in chicken macrophages. The similar replication kinetics of NDV strains CA02 and Anhinga/93 in DF1 cells and in lymphocytes (adherent splenocytes) indicate a macrophage-specific growth defect for NDV strain Anhinga/93 (Figs 1a, b and 2a, b).

### NDV F and HN proteins are important for its ability to replicate in macrophages

**Fig. 3.**
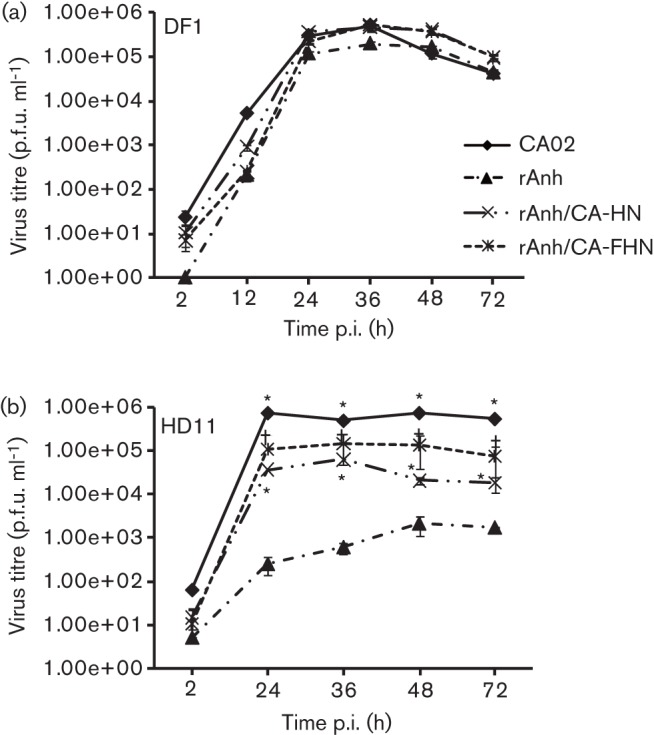
Replication kinetics of NDV strains CA02, rAnh, and chimeric viruses rAnh/CA-HN and rAnh/CA-FHN. (a) Multi-step growth curve in chicken fibroblasts (DF1; m.o.i. = 0.01). Cells were inoculated with each virus and harvested at 2, 12, 24, 36, 48 and 72 h p.i. Virus titres were determined by plaque assay and expressed as p.f.u. ml^−1^. (b) Multi-step growth curve in chicken macrophages (HD11; m.o.i. = 0.01). Cells were inoculated with each virus and harvested at 2, 24, 36, 48 and 72 h p.i. Virus titres were determined by plaque assay and expressed as p.f.u. ml^−1^. *P≤0.05 CA02, rAnh/CA-HN and rAnh/CA-FHN vs rAnh. Results shown are the mean±se of two (2 and 72 h p.i.) or three independent experiments.

**Fig. 4.**
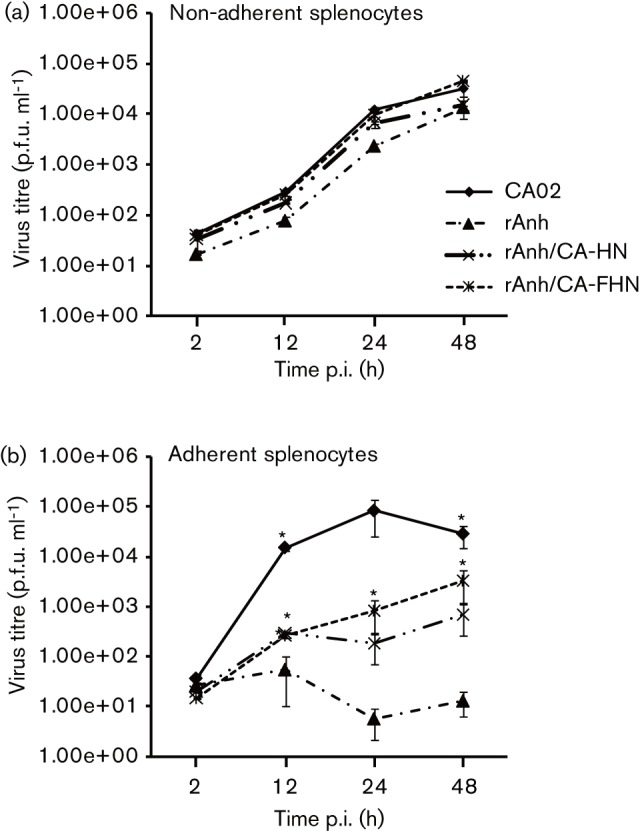
Replication kinetics of NDV strains CA02, rAnh, and chimeric viruses rAnh/CA-HN and rAnh/CA-FHN in primary splenocytes. Multi-step growth curve in chicken (a) lymphocytes (non-adherent splenocytes) and (b) macrophages (adherent splenocytes). Cells were inoculated with each virus (m.o.i. = 0.01) and harvested at 2, 12, 24 and 48 h p.i. Virus titres were determined by plaque assay and expressed as p.f.u. ml^−1^. *P≤0.05 CA02, rAnh/CA-HN and rAnh/CA-FHN vs rAnh. Results shown are the mean±se of three independent experiments.

Given the central role of the F and HN proteins for NDV infection, host range and tissue tropism (Dortmans *et al.*, 2011; Huang *et al*., 2004; Panda *et al.*, 2004), the importance of these proteins for NDV infection/replication in macrophages was investigated. HD11 cells were cultured in 12-well plates (10^4^ cells per well) and inoculated with NDV strains CA02, recombinant Anhinga/93 (rAnh) or with two chimeric viruses (rAnh/CA-HN and rAnh/CA-FHN) (m.o.i. = 0.01), in which the HN or the F and HN genes of NDV strain Anhinga/93 have been replaced by the corresponding genes from NDV strain CA02 (Estevez *et al.*, 2007). Viral titres were determined by plaque assay at 2, 24, 36, 48 and 72 h p.i. and expressed as p.f.u. ml^−1^. Similar to the results obtained with the wt NDV strain Anhinga/93 (Fig. 2a, b), the rAnh presented a marked decrease in the ability to replicate in macrophages, as evidenced by significant differences in replication kinetics and viral yields when compared with NDV strain CA02 (Fig. 3b). Notably, when the HN or both the F and HN genes of NDV rAnh were replaced by the corresponding genes from NDV strain CA02 a marked improvement in the ability of the chimeric viruses to replicate in HD11 cells was observed (Fig. 3b). Although the HN gene from NDV strain CA02 was sufficient to improve replication of the chimera rAnh/CA-HN, exchange of both F and HN genes in the rAnh/CA-FHN virus resulted in replication kinetics comparable to the parental virus (Fig. 3b). Similar results where observed when the replication kinetics of NDV CA02, rAnh, rAnh/CA-HN and rAnh/CA-FHN where compared in primary macrophages (adherent splenocytes; Fig. 4b). On the other hand, no differences in replication kinetics or viral where observed in control DF1 and non-adherent splenocyte cultures (Figs. 3a, 4a). Together these results indicate that the F and HN proteins contribute to NDV macrophage host range.

### The F and HN proteins of NDV strain Anhinga/93 present unique amino acid substitutions

Comparison of the amino acid sequence of the F and HN proteins of NDV strains CA02 and Anhinga/93 revealed 15 non-conserved amino acid changes on the F protein (R8W, I16T, T19I, M20T, G110R, N170D, N188D, A203T, D342N, T397I, S425N, S449L, T477A, N486S and A513V) and 12 non-conserved substitutions on the HN protein (A28T, E49G, T52I, N54S, N76S, S77L, S120N, A148V, T216I, T255I, H439Y and Q496R) (data not shown). Of these, three amino acid substitutions in the F protein (I16T, T397I and S449L) and three in the HN (T216I, H439Y and Q496R) are unique to NDV strain Anhinga/93, and are not present in any other strain used in this study (CA02, ZJ1, Peru/08, TX4156 and Nevada/05) (data not shown).

## Discussion

It has been demonstrated that the NDV surface proteins F and HN play critical roles in mediating infection of host cells, and consequently these proteins are important determinants of NDV tissue tropism and virulence (de Leeuw *et al.*, 2005; Huang *et al*., 2004; Panda *et al.*, 2004). Results here show that NDV strain Anhinga/93 has an impaired ability to replicate in chicken macrophages, when compared with NDV strain CA02. Despite differences in virulence in chickens, NDV strains CA02 and Anhinga/93 share the same multi-basic amino acid F protein cleavage site (^112^R-R-Q-K-R-F^117^). Therefore, the differences in growth characteristics presented by these strains in macrophages is probably not the result of an inefficient cleavage of the F protein. The similar replication kinetics of both strains in chicken fibroblasts and in lymphocytes (non-adherent splenocytes) further support this observation. Comparison of the amino acid sequence of the F and HN proteins revealed unique amino acid substitutions in NDV strain Anhinga/93 (see above). Although the importance of these specific amino acid residues for the functions of the F and HN proteins is unknown, individual or multiple changes in these residues may affect the ability of NDV to infect/replicate in macrophages.

The HN of NDV strain CA02 may improve viral infection and replication in chicken macrophages by increasing viral attachment and entry via sialylated cell surface molecules such as the mannose receptor. For example, for influenza virus, the haemagglutinin binds to macrophage mannose receptors mediating viral endocytosis and the avidity of the influenza haemagglutinin for macrophage mannose receptor correlates with viral particle uptake (Reading *et al.*, 2000). This process, however, does not lead to infection by influenza, because the virions fail to fuse with the macrophage cell membrane. In contrast to influenza virus, NDV does not require a reduced pH to induce cell membrane fusion, so binding and uptake via the macrophage mannose receptors may represent a viable method of cell infection (Markwell *et al.*, 1985). Alternatively, the HN of NDV strain CA02 may mediate an improved activation of the F protein, a necessary step in cell membrane fusion. This possibility is supported by the improved replication capability of rAnh/CA-FHN and corroborates the notion that the F and HN proteins from homologous viruses improve interaction and infection efficiency when compared with F and HN proteins from heterologous viruses (Gravel & Morrison, 2003; Huang *et al.*, 2004).

In summary, we have shown here that NDV strain Anhinga/93 presents an impaired ability to replicate in chicken macrophages when compared with the closely related NDV strain CA02. The infection/replication advantage of NDV strain CA02 results from the function of both surface glycoproteins, indicating that the F and HN are determinants of NDV macrophage host range. Replication in macrophages may represent an important advantage during infections *in vivo*, because these cells are present in a wide variety of host tissues, are highly motile cells, and are key mediators/modulators of the host immune response to viral infection (Abbas & Lichtman, 2005; Mogensen, 1979). A virus that is capable of replicating in host macrophages has access to multiple organ systems and can alter the host immune responses favouring viral replication.

## Methods

### 

#### Cells and viruses.

Chicken embryo fibroblasts (DF1, ATCC) were cultured in Dulbecco’s modified Eagle’s medium (Invitrogen) supplemented with 10 % FBS (Invitrogen) and antibiotics (100 U penicillin ml^−1^, 100 µg streptomycin ml^−1^, 0.25 µg amphotericin B ml^−1^; Thermo Scientific), and maintained at 37 °C with 5 % CO_2_. The chicken macrophage cell line HD11 was cultured in RPMI 1640 medium (Clontech) supplemented with 10 % FBS and antibiotics as above. Primary splenocyte cultures were obtained from SPF chickens. Ten chickens were euthanized, the spleens were removed aseptically, and splenocyte suspensions were generated by pushing the spleens through a sterile cell strainer into a 50 ml conical tube containing cell culture medium (RPMI 1640 with glutamine) supplemented with 10 % FBS and penicillin/streptomycin as above. The resulting cell suspension was layered on Ficoll and centrifuged at 500 ***g*** for 20 min. Cells were removed from the Ficoll gradient, resuspended in culture medium, washed twice and cultured in 12-well plates (10^4^ cells ml^−1^) for 24 h prior to virus infection.

The NDV strains CA02, ZJ1, Peru/08, Anhinga/93, Nevada/05 and TX4156 were obtained from the SEPRL repository and the recombinant NDV Anh (rAnh) and rAnh/CA-HN and rAnh/CA-FHN were kindly provided by Dr Qingzhong Yu (Estevez *et al.*, 2007). All viruses were propagated in 9 day-old SPF embryonated chicken eggs and the virus titres were determined by limiting dilution or plaque assay, calculated according to the Spearman and Karber method, and expressed as log_10_(TCID_50_ ml^−1^)or p.f.u. ml^−1^ as indicated. The recombinant chimeric viruses have been previously characterized as for their pathogenicity in chickens (Estevez *et al.*, 2007).

#### Growth curves.

The replication kinetics of NDV strains CA02, ZJ1, Peru/08, Anhinga/93, Nevada/05 and TX4156 (Diel *et al.*, 2012; Liu *et al.*, 2007; Wakamatsu *et al.*, 2006; Estevez *et al.*, 2007; Susta *et al.*, 2011) were investigated *in vitro*. Chicken fibroblast and macrophage cell lines (DF1 and HD11, respectively) were cultured in six-well plates (10^6^ cells per well) and inoculated with each virus at an m.o.i. of 0.01. The supernatant of infected cells was harvested at 0, 6, 12, 24, 36, 48 and 72 h p.i. and the virus titres were determined by limiting dilution, calculated according to the Spearman and Karber method, and expressed as log_10_(TCID_50_ ml^−1^).

The replication kinetics of NDV strains CA02 and Anhinga/93 were further investigated in primary chicken macrophages. Splenocytes were obtained from SPF chickens used as described above and three cell populations were separated: (1) total splenocytes; (2) non-adherent splenocytes (predominantly lymphocytes); and (3) adherent splenocytes (predominantly macrophages). Each cell type was cultured in 12-well plates (10^4^ cells per well) and inoculated with NDV strains CA02 and Anhinga/93 at an m.o.i. of 0.01. The supernatant of infected cells was harvested at 2, 12, 24 and 48 h p.i. and the virus titres were determined by plaque assay and expressed as p.f.u. ml^−1^ (Huang *et al.*, 2004).

To assess the role of the F and HN proteins on the ability of NDV to replicate in macrophages, replication kinetics of NDV strain CA02 and recombinant viruses rAnh, rAnh/CA-HN and rAnh/CA-FHN were investigated in HD11 and primary adherent splenocytes. DF1 cells and primary non-adherent splenocytes were used as controls. Cells were cultured in 12-well plates (10^4^ cells per well) and inoculated with NDV strains CA02, rAnh, rAnh/CA-HN and rAnh/CA-FHN at an m.o.i. of 0.01. The supernatant of infected cells was harvested at 2, 24, 36, 48 and/or 72 h p.i. (as indicated on graphs) and the virus titres were determined by plaque assay and expressed as p.f.u. ml^−1^ (Huang *et al.*, 2004). Statistical analysis was performed with JMP software (version 8) using the matched-pair analysis.

#### Sequence alignment.

Alignment and comparison of the amino acid sequences of NDV strains CA02 and Anhinga/93 were performed using mega5 and clustal w.

#### Accession numbers.

The complete amino acid sequences of the F and HN proteins of NDV strains CA02 and Anhinga/93 are available on GenBank under the accession numbers AAS67141, AAS67135 (F proteins), AAS67142, AAS67136 (HN proteins).
